# Thulium laser-assisted delivery of growth factors for treating androgenetic alopecia – a randomized, controlled, clinical study

**DOI:** 10.1007/s10103-026-05066-1

**Published:** 2026-10-22

**Authors:** Lynhda Nguyen, Stefan W. Schneider, Katharina Herberger

**Affiliations:** Department of Dermatology and Venereology, University Medical Center Hamburg-Eppendorf, Hamburg, Germany

**Keywords:** Hair loss, Androgenetic alopecia, Laser, Energy-based device, Growth factor

## Abstract

Androgenetic alopecia (AGA) is the most common form of hair loss and can significantly impact quality of life. Emerging non-invasive treatments include laser-assisted delivery of biomimetic growth factor–based serums. The aim of the study was to evaluate the safety and efficacy of non-ablative fractional laser (NAFL)–assisted delivery, with or without adjunct red light therapy, in patients with AGA. In this pilot study, patients were randomly assigned to: (I) NAFL-assisted serum delivery plus red light therapy (triple), (II) NAFL-assisted serum delivery alone (dual), or (III) NAFL plus red light therapy without serum (EBD-only). Primary outcomes were hair density and thickness. Secondary outcomes included patient satisfaction, tolerability, and adverse events. Hair density increased in all groups, with greater improvement in the triple (25.6 ± 7.0 %) and dual (22.7 ± 3.4 %) groups compared to EBD-only (4.9 ± 9.2 %). Hair thickness improved in both serum groups but not in the EBD-only group. Clinical outcomes were similar between triple and dual treatments, though satisfaction was higher with triple therapy. No severe adverse events occurred. Preliminary evidence suggests that NAFL-assisted delivery of biomimetic growth factors is a safe and effective non-invasive treatment for AGA.

**Supplementary Information:**

The online version contains supplementary material available at https://doi.org/10.1007/s10103-026-05066-1.

## Introduction

Androgenetic alopecia (AGA) is the most prevalent form of hair loss, affecting both men and women [1, 2]. It is typically marked by a progressive recession of the frontal hairline and thinning at the vertex in men, while women often experience diffuse thinning over the crown of the scalp [3, 4]. Although the exact pathogenesis remains unclear, AGA is thought to be influenced by genetic predisposition and androgen-dependent mechanisms [5, 6]. The condition varies widely in terms of age of onset, severity, and pattern of hair loss [2]. AGA affects approximately 30% of women over the age of 50 and up to 66% of men aged 55 and older [7, 8].

As hair is associated with self-image, AGA can have a significant emotional impact, often leading to a reduced quality of life [9]. Hair loss, particularly when progressive and visible, may cause distress, lower self-esteem, and depression [9]. Given its chronic and visible nature, there is a pressing need for treatment [10].

Although AGA is a common condition encountered in dermatological practice, it can be challenging to treat effectively [11, 12]. Based on a meta-analysis of randomized controlled studies (RCTs), minoxidil, finasteride, and red light stimulation are currently recognized as effective treatment options for AGA [11]. However, their benefits are often transient and diminish upon discontinuation of treatment [13]. In recent years, a novel therapeutic approach has been introduced into clinical practice, combining laser-assisted delivery of biomimetic growth factors that replicate naturally occurring human growth factors and peptides. These bioactive compounds are encapsulated within nano-liposomes to enhance transdermal penetration and are thought to be photoactivated by red light therapy, potentially augmenting their biological efficacy. Despite promising eminence, clinical data supporting this novel approach remain limited. Therefore, the present pilot study aimed to assess the safety and efficacy of laser-assisted delivery of biomimetic growth factors in the treatment of androgenetic alopecia.

## Materials and methods

### Study design

This pilot study was designed as a randomized, controlled, evaluator-blinded, clinical study. It was in accordance with the Declaration of Helsinki. It was retrospectively registered on ClinicalTrials.gov (NCT07079657). Male and female patients with AGA diagnosed by a board-certified dermatologist were recruited at the Laser Department of the University Medical-Center Hamburg-Eppendorf, Hamburg, Germany. After informed consent, patients were randomly assigned to one of the following three treatment arms: the triple group: Thulium (Tm) laser-assisted delivery of a growth factor serum and subsequent red light treatment; the dual group: Tm laser-assisted application of a growth factor serum, or the energy-base device (EBD) treatment-only group: Tm laser with subsequent red light treatment. Patients received three treatment sessions at intervals of 6 weeks. Patients who received the triple or dual treatment additionally received a home-kit consisting of a growth factor solution as well as a conditioning shampoo. For summary, see Table 1. Follow-up visits were scheduled 1 and 6 months post-treatment. Patients were instructed to stop any other topical or systemic treatment for hair loss throughout the period of the study up to the follow-up visits. Due to the nature of the interventions, blinding of the patients was not feasible. Outcome assessments were performed by two investigators blinded to group allocation.

**Table 1:** Overview of study groups

	1927 nm thulium laser treatment	Growth factor serum	red light therapy	home-kit (growth factor solution + shampoo)
Group A	✓	✓	✓	✓
Group B	✓	✓	x	✓
Group C	✓	x	✓	x

### Randomization method

Patients were randomly assigned to the treatment groups in a 1:1:1 ratio. Allocation concealment was ensured by using a sequentially numbered list based on a computer-generated randomization. 

### Inclusion and exclusion criteria

Inclusion criteria were patients at the age of 18 years or older. Exclusion criteria were pregnancy, nursing, history of skin cancer of the head, or surgical treatment on the area to be treated in the last 2 months. Patients with hair transplantation in the last 2 months were also excluded.

### Treatment protocol

Laser treatment was performed using a 1927 nm Tm laser (LaseMD Ultra^®^, Cynosure Lutronic^®^, Seoul, Korea) with the following parameters: C1 cap/ comb tip, static mode, 20 W, 3 mJ, two passes. To ensure full scalp coverage, the hair was parted into uniform sections. Following the laser procedure, if assigned, patients received an in-office application of a growth factor serum (in-office serum, Kerafactor^®^, Santa Monica, CA, USA), which was gently massaged into the scalp. Subsequently, if indicated, patients underwent red light therapy (Healite II, Cynosure Lutronic^®^, Seoul, Korea) using the following parameters: 633 nm, intensity 1, 40 J/cm^2^, 10 min per side.

Patients who received the growth factor serum were instructed to use a home care kit consisting of a daily scalp application of the growth factor solution (Scalp Stimulating Solution, Kerafactor^®^, Santa Monica, CA, USA) and the corresponding shampoo (Scalp Stimulating Shampoo, Kerafactor^®^, Santa Monica, CA, USA). Each kit provided approximately two weeks of use.

### Standardized photo documentation

Photographic documentation was performed using a hair growth analysis system (GroTrack^®^, Gro Technologies, Seattle, WA, USA). To ensure consistent positioning, all images were taken with standardized lighting provided by a single ring light. Patients were seated upright, and the hair at the crown was centrally parted before each session to ensure comparability between visits. Photographs were taken from the top of the head. For hair growth assessment, close-up images were taken of an area of 0.8 cm^2^. For consistent positioning of close-ups across all visits, a ruler was used to define three fixed measurement points – 3 cm, 5 cm, and 8 cm – from the frontal hairline along the central part.

### Assessment of hair density and hair diameter

High-resolution scalp images were captured at baseline, 1 month, and 6 months post-treatment. Hair density and shaft diameter were manually quantified using ImageJ software (National Institutes of Health, USA) [14]. For both hair density and diameter of hair shafts, three predefined scalp regions were assessed at each time point. Evaluation was conducted by two blinded investigators.

### Clinical assessment and patient satisfaction

Clinical evaluations were conducted by a blinded investigator at 1 and 6 months following the final treatment session. The overall clinical outcome was assessed using a 7-point scale ranging from + 3 (much improved) to -3 (much worsened). Additionally, patients were asked to rate their satisfaction with the treatment outcome on a 6-point Likert scale, from 1 (very satisfied) to 6 (not at all satisfied).

### Safety and tolerability

All adverse events (AEs), including edema, pruritus, and dyspigmentation, were documented and treated as appropriate. During each treatment session, patients rated their pain using the Numeric Rating Scale (NRS), ranging from 0 (no pain) to 10 (unbearable pain). Furthermore, patients were asked to evaluate their overall tolerability of the procedure on a 6-point scale from 1 (very well tolerated) to 6 (not at all tolerated).

### Statistical analysis

Statistical evaluation was performed using GraphPad Prism (Version 10.2.3, GraphPad Software Inc.). If not stated otherwise, the nonparametric Kruskal-Wallis analysis of variance (ANOVA) was used to determine the difference in medians between groups, followed by Dunn’s post-hoc test for multiple pairwise comparisons. For longitudinal, paired within-group comparisons, the non-parametric Wilcoxon signed-rank test was applied. P-values < 0.05 were defined as significant. Descriptive data were presented as means, standard deviations, and ranges (minimum – maximum) or medians and their interquartiles.

## Results

### Baseline characteristics

From October 2024 to July 2025, a total of 10 patients were enrolled in each of the three treatment groups. In the dual group, one patient was lost to follow-up, and in the EBD-only group, two patients were lost to follow-up. No patients discontinued the study due to AEs. The grade of hair loss was assessed using the Ludwig and Norwood-Hamilton grade for female and male patients, respectively. The groups were statistically comparable at baseline (*p* > 0.05). Details are presented in Table 2.

**Table 2:** Patient characteristics

	Triple group	Dual group	EBD-only group
Sex (female/ male)	8/ 2	7/ 3	6/ 4
Age (mean ± SD (min. – max.))	47.4 ± 7.2 (29–54)	47.5 ± 11.0 (34–61)	53.4 ± 12.0 (33–65)
Ludwig grade (median (interquartile range)) Norwood-Hamilton grade (median (interquartile range))	1.5 (1; 2) 3 (3; 3)	1 (1; 2) 3 (3; 3.5)	1 (1; 1.75) 3.5 (3; 4)

### Hair density

**Fig. 1:**
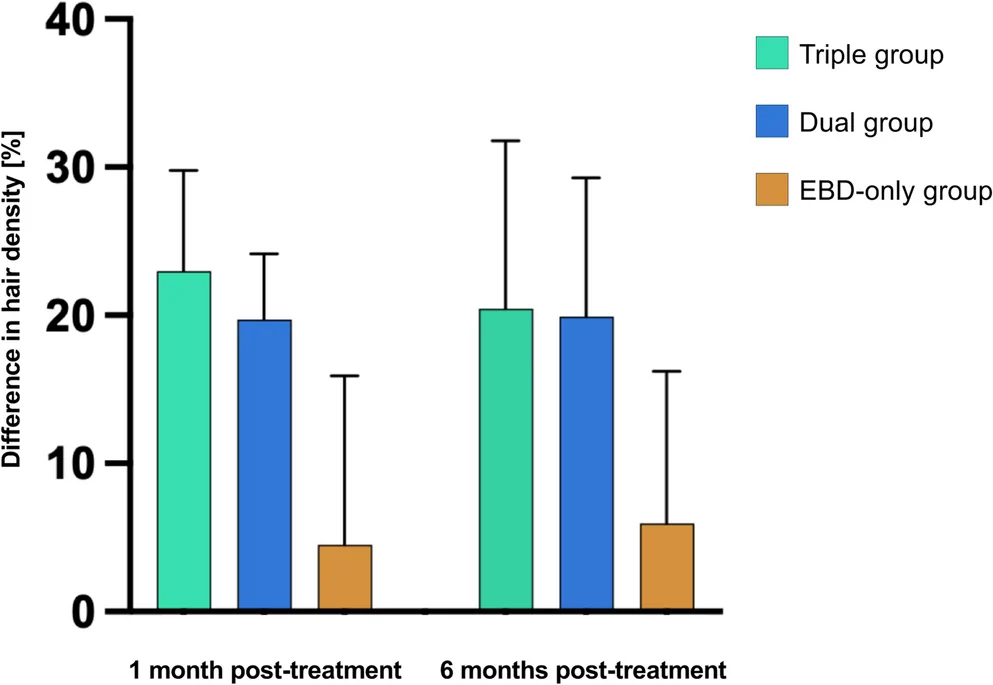
Difference in hair density after 1 and 6 months post-treatment across all treatment groups

At 1 month post-treatment, all three treatment groups demonstrated an increase in hair density compared to baseline, though the level of change differed (Fig. 1).

Mean hair density increased by 25.6 ± 7.0% (19.7–37.2, *p* < 0.05 vs. baseline) in the triple group, 22.7 ± 3.4% (19.2–25.9, *p* < 0.05 vs. baseline) in the dual group, and 4.9 ± 9.2% (-23.8–13.2, *p* < 0.05 vs. baseline) in the EBD-only group. Post-hoc pairwise comparisons confirmed that hair density increases in the triple and dual groups were significantly greater than in the EBD-only group (*p* < 0.05 for both), while there was no evidence of a difference between the triple and dual groups (*p* > 0.05).

At 6 months post-treatment, hair density remained stable in both combination groups relative to 1 month. The triple group maintained a mean increase of 22.3 ± 13.6% (9.6–42.9, *p* > 0.05 vs. 1 month; *p* < 0.05 vs. baseline), and the dual group maintained 22.3 ± 10.3% (11.2–35.7, *p* > 0.05 vs. 1 month; *p* < 0.05 vs. baseline). The EBD-only group showed a modest but still distinct improvement of 7.0 ± 10.5% (-22.0–22.3, *p* > 0.05 vs. 1 month, *p* < 0.05 vs. baseline). Between-group differences at 6 months remained significant: Dunn’s post-hoc test showed that hair density in both the triple and dual groups was higher than in the EBD-only group (*p* < 0.05 for both), with no clear evidence of a difference between the triple and dual groups (*p* < 0.05).

### Diameter of hair shaft

**Fig. 2:**
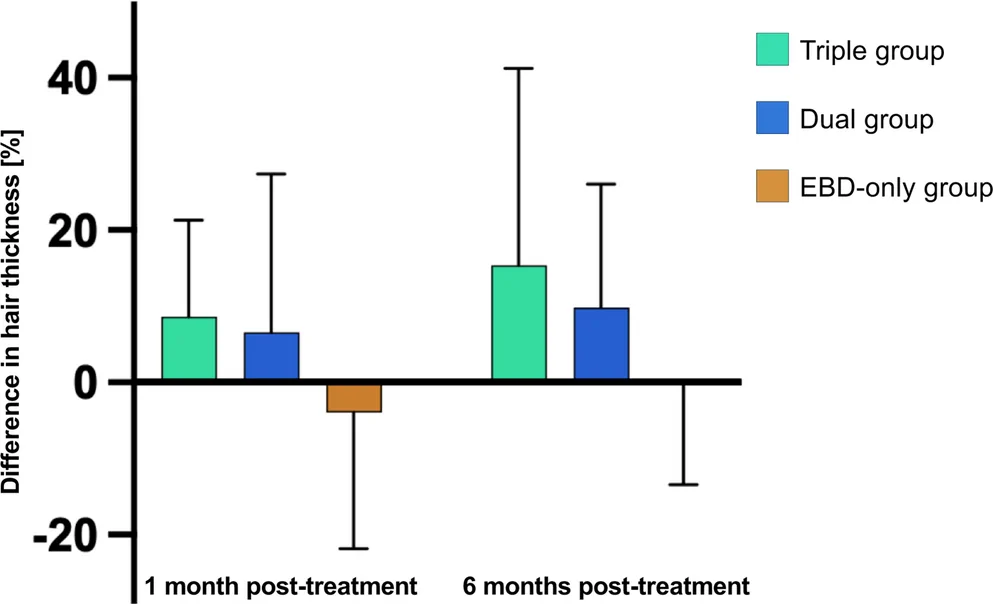
Difference in hair thickness after 1 and 6 months post-treatment across all three study groups

At 1 month post-treatment, all three groups demonstrated changes in hair thickness compared with baseline (Fig. 2). The triple group showed a significant increase of 9.4 ± 15.8% (-22.6–17.8, *p* < 0.05 vs. baseline) and the dual group had an increase of 8.3 ± 17.0% (-24.4–11.1, *p* < 0.05 vs. baseline). In contrast, the EBD-only group experienced a decrease in hair thickness of 3.8 ± 21.6% (-22.5–33.9, *p* < 0.05 vs. baseline). Dunn’s post-hoc test confirmed that the triple and dual groups improved significantly more than the EBD-only group (*p* < 0.05 for both).

At 6 months post-treatment, hair thickness continued to improve in the combination therapy groups. The triple group increased by 13.8 ± 26.1% (-17.3–44.4, *p* < 0.05 vs. baseline; *p* > 0.05 vs. 1 month), and the dual group increased by 11.9 ± 18.0% (-13.8–34.3, *p* < 0.05 vs. baseline; *p* > 0.05 vs. 1 month). The EBD-only group showed minimal further change (–0.7 ± 12.8%; -12.6–16.4; *p* > 0.05 vs. baseline; *p* > 0.05 vs. 1 month). Between-group comparisons showed clear differences: both triple and dual groups were higher than EBD-only (*p* < 0.05), with no evidence of a difference between triple and dual groups (*p* > 0.05).

### Clinical assessment

At 1 month post-treatment, both the triple and dual groups demonstrated clinical improvement compared with baseline, with mean scores of 1.8 ± 1.0 (1–3) and 1.8 ± 0.5 (1–2), respectively. No measurable difference was observed between the triple and dual treatment groups (*p* > 0.05). In contrast, the EBD-only group showed minimal improvement, with a mean score of 0.3 ± 0.6 (0–1).

**Fig. 3:**
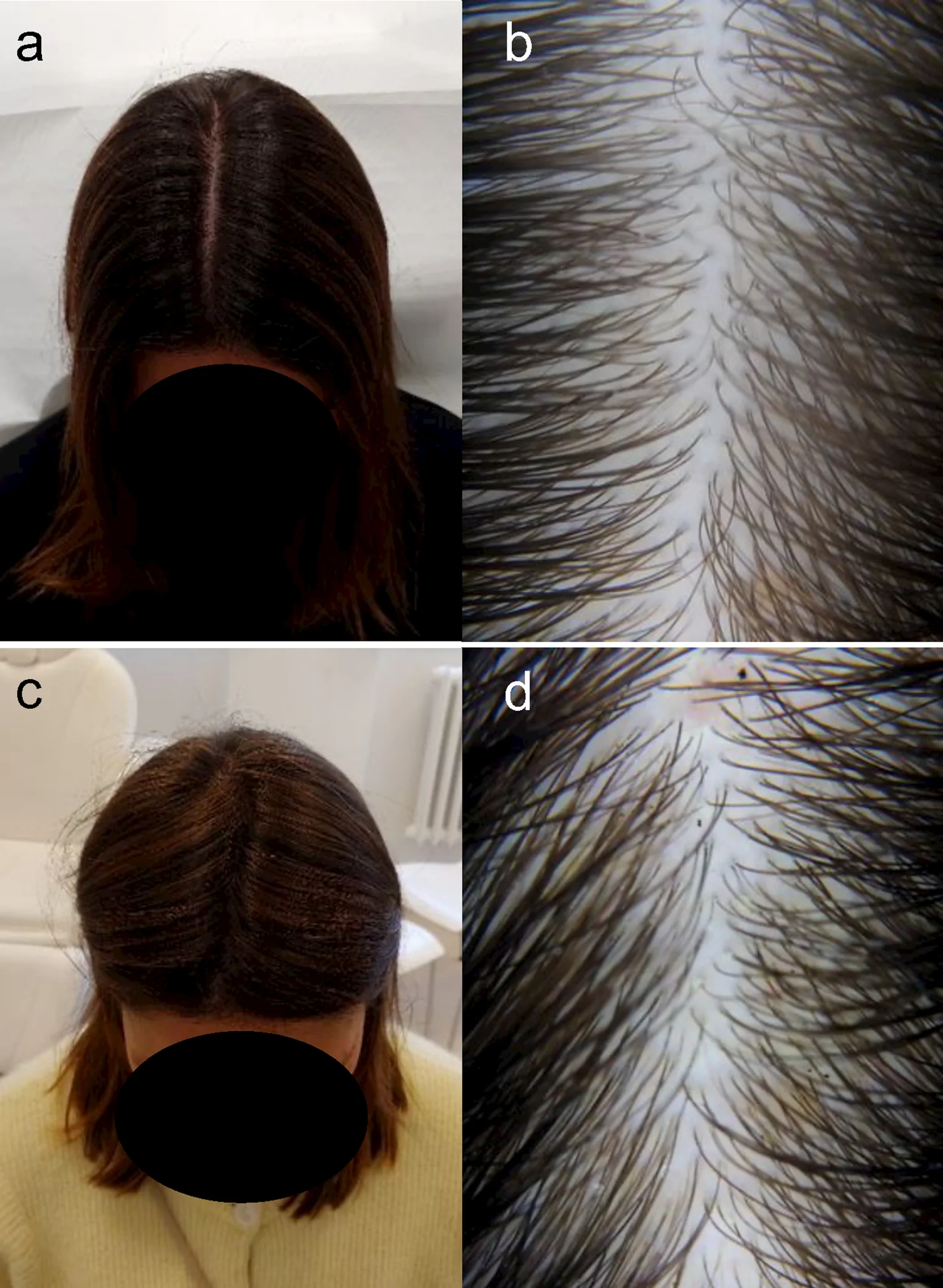
Clinical and close-up images of a patient from the triple group (**a**,** b**) before and (**c**,** d**) 6 months after the final treatment session. The patient experienced a marked improvement in hair density and thickness

**Fig. 4:**
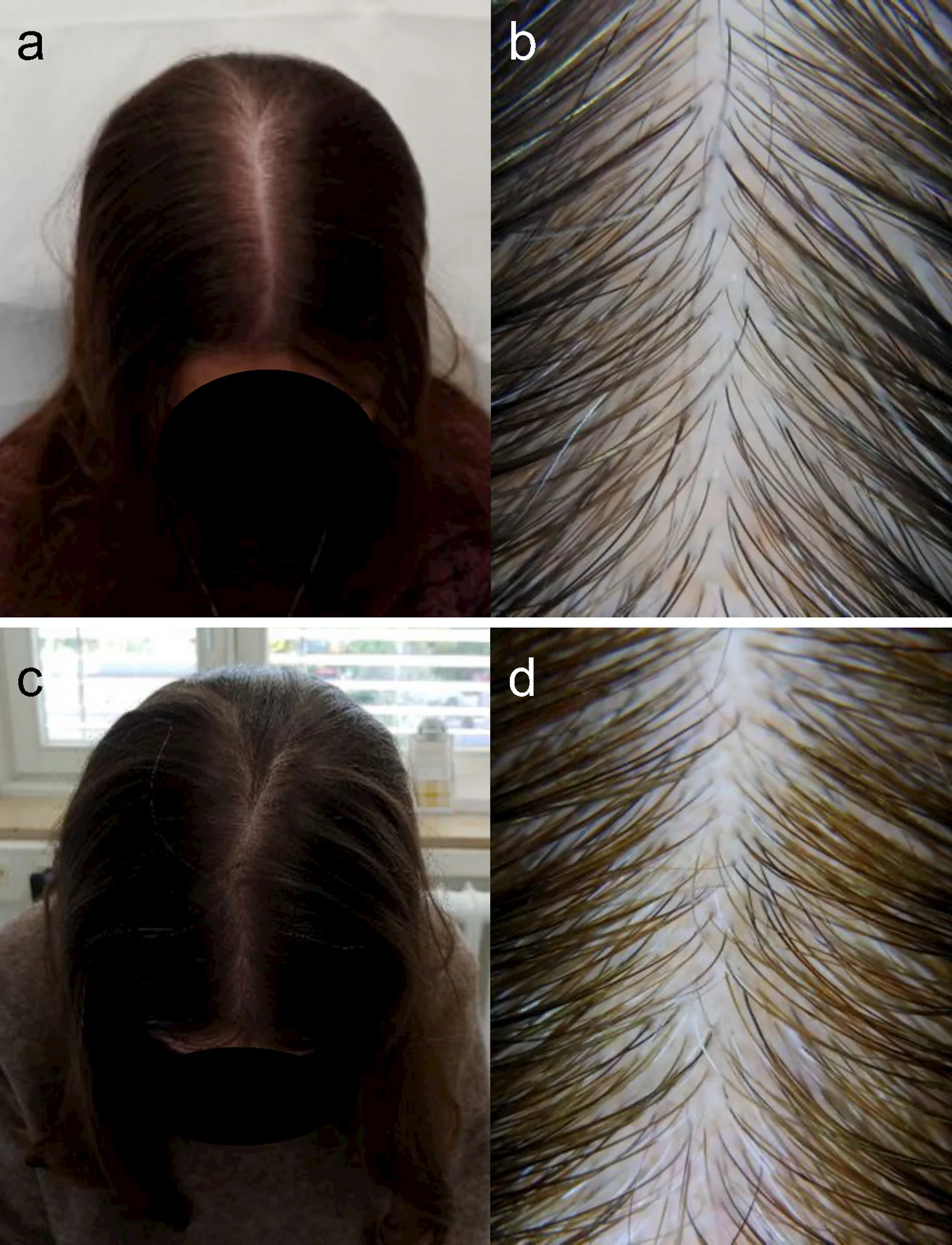
Baseline (**a**,** b**) and six-month follow-up (**c**,** d**) clinical and close-up images of a patient from the dual group. Noticeable increases in hair density and thickness were observed following treatment

**Fig. 5:**
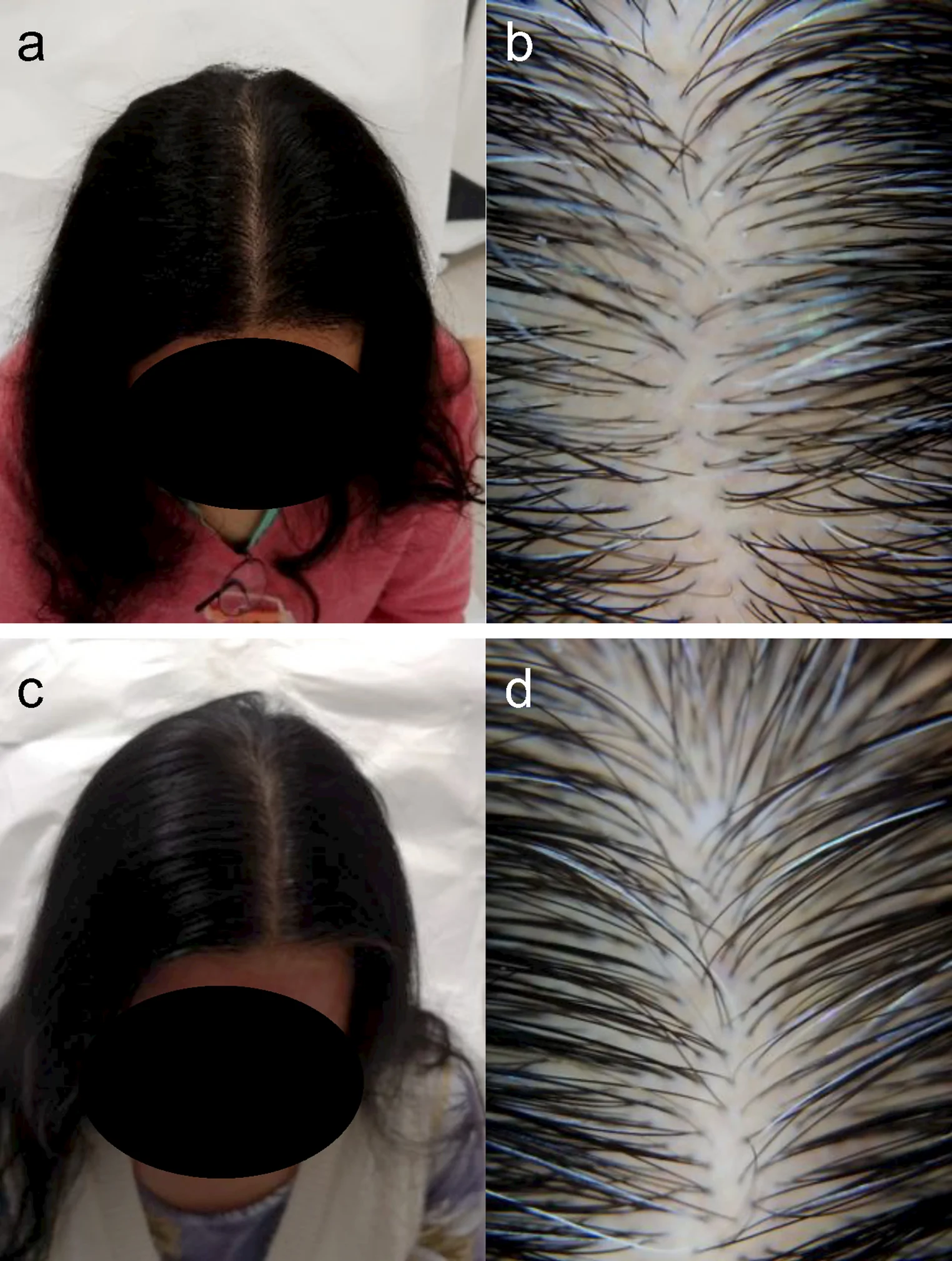
Clinical and close-up views of a patient in the EBD-only group (**a**,** b**) at baseline and (**c**,** d**) 6 months after the final treatment session. Patient experienced a mild improvement of her androgenetic alopecia

At 6 months post-treatment, sustained improvement was observed in both the triple and dual groups, with mean scores of 1.5 ± 1.3 (0–3) and 1.3 ± 1.0 (0–3), respectively. No measurable difference in improvement was detected between these groups. The EBD-only group continued to demonstrate only slight improvement, with a mean score of 0.3 ± 0.5 (0–1). Figures 3 and 4, and Fig. 5 show representative clinical outcomes 6 months after the last treatment session.

### Patient satisfaction

Regarding patient satisfaction, the mean satisfaction score at 1 month post-treatment was 1.3 ± 0.5 (1–2) in the triple group, 2.3 ± 1.9 (1–5) in the dual group, and 3.3 ± 0.5 (3–4) in the EBD-only group, showing a statistically significant difference between the groups (*p* < 0.05).

At 6 months post-treatment, satisfaction scores did not differ significantly compared with those at 1 month (*p* > 0.05). The mean scores were 1.9 ± 1.6 (1–5) for the triple group, 2.5 ± 1.6 (1–5) for the dual group, and 2.9 ± 1.1 (1–4) for the EBD-only group.

### Safety and tolerability

Across all treatment groups, patients reported high tolerability. Mean pain intensity scores were 0.4 ± 0.5 (0–1) in the triple group, 0.3 ± 0.5 (0–1) in the dual group, and 1.0 ± 1.3 (0–4) in the EBD-only group which did not show a statistical significance between groups (*p* > 0.05). Two patients (2/10) in the EBD-only group experienced mild, transient itchiness and dandruff following treatment, which resolved spontaneously after a few days. No treatment-related serious AEs were reported during the study period.

## Discussion

The present pilot study evaluated the efficacy and safety of a combination therapy involving laser-assisted delivery of a biomimetic growth factor and peptide-based serum in conjunction with red light treatment in patients with AGA. To the best of our knowledge, this is the first RCT to directly investigate this multimodal therapeutic approach for AGA.

Although the exact pathophysiological mechanisms of AGA is not fully understood, the condition is widely recognized as polygenic and androgen-dependent [5, 6]. AGA is characterized by a progressive miniaturization of hair follicles, resulting in the vellus transformation of terminal hairs [15]. This process is driven by alterations in hair cycle dynamics, where the anagen phase becomes progressively shortened while the telogen phase is prolonged [16, 17]. Because the duration of the anagen phase determines the potential hair length, this imbalance leads to the production of shorter and thinner hair shafts, and over time, a reduction in hair density and thickness [16, 17].

A wide range of therapeutic options is currently available for the management of AGA, including topical pharmacologic agents, systemic therapies, and EBDs such as LLLT and red light therapy [9]. Despite these diverse options, AGA management remains challenging, as current treatments often produce variable outcomes, require long-term adherence, and tend to lose effectiveness upon discontinuation [9]. Consequently, there is an increasing interest in complementary therapies.

Fractional NAFLs, like the 1927 nm Tm laser utilized in this study, have demonstrated efficacy in facilitating transdermal drug delivery with minimal downtime [18–20]. The fractional laser creates controlled microchannels, temporarily disrupting the skin barrier while preserving tissue integrity when applied at low fluence [21]. The accompanying serum with the KFP™ formula contains synthetic peptides and growth factors that mimic naturally occurring signalling molecules involved in hair follicle induction. By targeting these molecular pathways, the therapy aims to stimulate dormant follicles, encourage the transition from the telogen to the anagen phase, and support the formation of new follicles.

In this study, the multimodal protocol with combined in-office and home-based therapy demonstrated both clinical efficacy and a favorable safety profile, with observed improvements in hair density and thickness following treatment. Patients reported minimal to no discomfort during the procedure, and no severe AEs occurred during the study. Furthermore, hair density remained stable six months after the final treatment session, suggesting sustained therapeutic benefits. These findings indicate that laser-assisted delivery of biomimetic growth factors combined with home-based therapy may represent a promising, non-invasive alternative for patients with AGA, particularly for those seeking treatments beyond conventional pharmacologic options such as finasteride or minoxidil.

Current literature supports the effectiveness of established therapies, although reported improvements vary across studies. In an RCT, topical minoxidil applied twice weekly led to a 13.4% increase in hair count by week 16 [22]. For oral finasteride, a systematic review demonstrated a long-term increase in hair count of approximately 24.3% compared with baseline [23]. Another systematic review including six studies reported a mean increase of 17.9 hairs/ cm² following treatment with platelet-rich plasma [24]. However, direct comparative studies and studies on combination treatments remain necessary to define its role in AGA treatment.

No evidence in a difference in hair density and thickness was detected between the triple group and the dual group, suggesting that the addition of the red light therapy did not further enhance clinical efficacy under the tested conditions. While red light therapy is believed to improve cellular metabolism and microcirculation, its additive impact may depend on factors such as settings and patient characteristics [25, 26]. Future studies should investigate these parameters further and include larger cohorts and extended follow-up periods to better characterize the synergistic potential of combining laser-assisted drug delivery with red light therapy in AGA treatment. Interestingly, patients in the triple group reported higher satisfaction scores, which may be attributed to the subjective comfort and warmth associated with the red light application.

The range of improvement observed across patients was variable. Patient-specific factors such as age, gender, grade of hair loss, hormonal status, genetic predisposition, and lifestyle may influence treatment response. Due to the small sample size, however, a subgroup analysis to evaluate these influences was not feasible.

Several limitations should be acknowledged. Due to a lack of a formal power calculation and the small sample size, statistical power and generalizability are limited. These results should be interpreted as preliminary. The relatively short follow-up duration may not fully reflect long-term treatment efficacy and sustainability. In addition, the use of combination treatment arms limits the ability to fully isolate the individual effects of each modality. However, the smaller improvement in the EBD-only group suggests that the observed benefit may be primarily attributable to the growth factor component. The absence of a specific measure of adherence to the home-based regimen may constitute a limitation. Moreover, the molecular mechanisms underlying the observed effects remain to be clarified. Future research should aim to validate these findings in larger trials with extended observation periods and incorporate histological and molecular analyses to better elucidate the biological pathways involved.

In conclusion, the present study provides promising evidence supporting the efficacy and safety of the laser -assisted delivery of biomimetic growth factors and peptide-based serum combined with home-based therapy for the management of AGA. Future studies with larger cohorts and extended follow-up periods are warranted to validate these findings, optimize treatment protocols, and identify patient-specific factors that may enable a more personalized therapeutic approach.

## Supplementary Information

Below is the link to the electronic supplementary material.

Supplementary Material 1.

Supplementary Material 2.

## Data Availability

The data that support the findings of this study are available from the corresponding author upon reasonable request.

## Declarations

### Clinical Trial registration

Registration number NCT07079657, registered in clinicaltrials.gov, date of registration 23 July 2025.

## References

[1] Severi G, Sinclair R, Hopper J et al (2003) Androgenetic alopecia in men aged 40–69 years: prevalence and risk factors. Br J Dermatol 149(6):1207–1213 10.1111/j.1365-2133.2003.05565.x 14674898

[2] Lolli F, Pallotti F, Rossi A et al (2017) Androgenetic alopecia: a review. Endocrine 57(1):9–17 10.1007/s12020-017-1280-y 28349362

[3] Starace M, Orlando G, Alessandrini A, Piraccini BM (2020) Female Androgenetic Alopecia: An Update on Diagnosis and Management. Am J Clin Dermatol 21(1):69–84 10.1007/s40257-019-00479-x 31677111

[4] Kelly Y, Blanco A, Tosti A (2016) Androgenetic alopecia: an update of treatment options. Drugs 76(14):1349–1364 10.1007/s40265-016-0629-5 27554257

[5] Rebora A (2004) Pathogenesis of androgenetic alopecia. J Am Acad Dermatol 50(5):777–779 10.1016/j.jaad.2003.11.073 15097964

[6] Trüeb RM (2002) Molecular mechanisms of androgenetic alopecia. Exp Gerontol 37(8–9):981–990 10.1016/s0531-5565(02)00093-1 12213548

[7] O’tar T (2001) Incidence of female androgenetic alopecia (female pattern alopecia). Dermatol Surg 27(1):53–54 11231244

[8] Severi G, Sinclair R, Hopper J et al (2003) Androgenetic alopecia in men aged 40–69 years: prevalence and risk factors. Br J Dermatol 149(6):1207–1213 10.1111/j.1365-2133.2003.05565.x 14674898

[9] Huang C-H, Fu Y, Chi C-C (2021) Health-related quality of life, depression, and self-esteem in patients with androgenetic alopecia: a systematic review and meta-analysis. JAMA dermatology 157(8):963–970 10.1001/jamadermatol.2021.2196 PMC8264758 34232264

[10] Kaiser M, Abdin R, Gaumond SI et al (2023) Treatment of androgenetic alopecia: current guidance and unmet needs. Clin Cosmet Investig Dermatol, 16:1387–1406 10.2147/CCID.S385861 PMC10239632 37284568

[11] Adil A, Godwin M (2017) The effectiveness of treatments for androgenetic alopecia: a systematic review and meta-analysis. J Am Acad Dermatol 77(1):136–141 e135 10.1016/j.jaad.2017.02.054 28396101

[12] Varothai S, Bergfeld WF (2014) Androgenetic alopecia: an evidence-based treatment update. Am J Clin Dermatol 15(3):217–230 10.1007/s40257-014-0077-5 24848508

[13] Nestor MS, Ablon G, Gade A et al (2021) Treatment options for androgenetic alopecia: Efficacy, side effects, compliance, financial considerations, and ethics. J Cosmet Dermatol 20(12):3759–3781 10.1111/jocd.14537 PMC9298335 34741573

[14] Schneider CA, Rasband WS, Eliceiri KW (2012) NIH Image to ImageJ: 25 years of image analysis. Nat Methods 9(7):671–675 10.1038/nmeth.2089 PMC5554542 22930834

[15] Whiting DA (2001) Possible mechanisms of miniaturization during androgenetic alopecia or pattern hair loss. J Am Acad Dermatol 45(3):S81–S86 10.1067/mjd.2001.117428 11511857

[16] Paus R, Cotsarelis G (1999) The biology of hair follicles. N Engl J Med 341(7):491–497 10.1056/NEJM199908123410706 10441606

[17] Katzer T, Leite Junior A, Beck R, da Silva C (2019) Physiopathology and current treatments of androgenetic alopecia: going beyond androgens and anti-androgens. Dermatol Ther 32(5):e13059 10.1111/dth.13059 31400254

[18] Nguyen L, Mess C, Schneider SW et al (2025) Multiphoton tomographic analysis of hyaluronic acid delivery: comparison of carbon dioxide laser and 1927 nm thulium laser over time. Lasers Med Sci 40(1):100 10.1007/s10103-025-04363-5 PMC11832699 39960562

[19] Wang JV, Christman MP, Feng H et al (2021) Laser-assisted delivery of tranexamic acid for melasma: Pilot study using a novel 1927 nm fractional thulium fiber laser. J Cosmet Dermatol 20(1):105–109 10.1111/jocd.13817 33174686

[20] Wang JV, Lopez A, Geronemus RG (2022) Safety and effectiveness of low-energy, low-density 1927-nm fractional thulium fiber laser with tranexamic acid for postinflammatory hyperpigmentation. Dermatol Surg 48(10):1131–1133 10.1097/DSS.0000000000003591 36037092

[21] Wenande E, Jacobsen K, Grove GL et al (2025) Imaging 1927 nm Fractional Thulium Laser-Tissue Interactions: A Spectrum of Nonablative to Ablative Effects. J Cosmet Dermatol 24(7):e70304 10.1111/jocd.70304 PMC12264580 40665805

[22] Olsen EA, Whiting D, Bergfeld W et al (2007) A multicenter, randomized, placebo-controlled, double-blind clinical trial of a novel formulation of 5% minoxidil topical foam versus placebo in the treatment of androgenetic alopecia in men. J Am Acad Dermatol 57(5):767–774 10.1016/j.jaad.2007.04.012 17761356

[23] Mella JM, Perret MC, Manzotti M et al (2010) Efficacy and safety of finasteride therapy for androgenetic alopecia: a systematic review. Arch Dermatol 146(10):1141–1150 10.1001/archdermatol.2010.256 20956649

[24] Giordano S, Romeo M, Lankinen P (2017) Platelet-rich plasma for androgenetic alopecia: Does it work? Evidence from meta analysis. J Cosmet Dermatol 16(3):374–381 10.1111/jocd.12331 28296142

[25] Lanzafame RJ, Blanche RR, Chiacchierini RP et al (2014) The growth of human scalp hair in females using visible red light laser and LED sources. Lasers Surg Med 46(8):601–607 10.1002/lsm.22277 PMC4265291 25124964

[26] Avci P, Gupta GK, Clark J et al (2014) Low-level laser (light) therapy (LLLT) for treatment of hair loss. Lasers Surg Med 46(2):144–151 10.1002/lsm.22170 PMC3944668 23970445

