# Supplementary material for: Thulium laser-assisted delivery of growth factors for treating androgenetic alopecia – a randomized, controlled, clinical study
Source: Lasers Med Sci. 2026 Oct 22;41(1):271. doi: 10.1007/s10103-026-05066-1 (PMC13690880; doi:10.1007/s10103-026-05066-1)
Supplement: Supplementary file 2 [file 10103_2026_5066_MOESM2_ESM.doc]

Sample template for the CONSORT diagram showing the flow of participants through each stage of a randomized trial.

Assessed for eligibility

(n = 30)


**Enrollment**

Excluded (n = 0)

Not meeting inclusion criteria

(n = 0)

Refused to participate

(n = 0)

Other reasons (n = 0)

Randomized (n = 30)


**Allocation**

**Follow up**

**Analysis**

Allocated to triple group

(n = 10)

Received allocated intervention (n = 10)

Did not receive allocated intervention (n = 0)

(give reasons)

Lost to follow up

(n = 0) (give reasons)

Discontinued intervention (n = 0) (give reasons)

Analyzed (n = 10)

Excluded from analysis

(n = 0) (give reasons)

Allocated dual group

(n = 10)

Received allocated intervention (n = 10)

Did not receive allocated intervention (n = 0)

(give reasons)

Lost to follow up

(n = 1) (personal reasons)

Discontinued intervention (n = 0) (give reasons)

Analyzed (n = 9)

Excluded from analysis

(n = 1) (lost to follow up)

Allocated EBD-only group

(n = 10)

Received allocated intervention (n = 10)

Did not receive allocated intervention (n = 0)

(give reasons)

Lost to follow up

(n = 2) (personal reasons)

Discontinued intervention (n = 0) (give reasons)

Analyzed (n = 8)

Excluded from analysis

(n = 2) (lost to follow up)
